# Supplementary material for: Association of fish intake with menstrual pain: A cross-sectional study of the Japan Environment and Children’s Study
Source: PLoS One. 2022 Jul 21;17(7):e0269042. doi: 10.1371/journal.pone.0269042 (PMC9302766; doi:10.1371/journal.pone.0269042)
Supplement: S1 Table — (PDF) [file pone.0269042.s001.pdf]

S1 Table. Covariates setting and measurement time

Covariates	Measurement time	Setting
Age	1.5 years after delivery	
Body mass index (BMI)	6 months after delivery	BMI was calculated from the height and body weight.
Smoking habit	1.5 years after delivery	
Passive smoking	1.5 years after delivery	
Alcohol intake	1.5 years after delivery	
Maternal educational level	24–28 weeks of gestation	The educational level was defined by the highest academic achievement.
Paternal educational level	24–28 weeks of gestation	The educational level was defined by the highest academic achievement.
Employment	1 years after delivery	Students were treated as workers
Family income	12–16 weeks of gestation	Family income was defined by the annual revenue of the household.
Marital status	12–16 weeks of gestation	
Parity	12–16 weeks of gestation	
Fetal number	from the birth charts	The birth charts were filled out by the doctor or midwife.
Mode of delivery	from the birth charts	The birth charts were filled out by the doctor or midwife.
Obstetric complication	from the birth charts	The birth charts were filled out by the doctor or midwife. Obstetric complications included threatened abortion, threatened premature delivery, gestational diabetes mellitus, hypertensive disorders of pregnancy, premature rupture of the membranes, placenta previa, non-reassuring fetal status, and placental abruption that women experienced during pregnancy.
Age at menarche	12–16 weeks of gestation	
History of gynecological disease	enrollment	The medical records were transcribed by physicians, midwives, nurses, or research coordinators. Gynecological disease included uterine fibroids, endometriosis, adenomyosis, and uterine malformations.

History of mental illness	enrollment	The medical records were transcribed by physicians, midwives, nurses, or research coordinators. A past history of mental illness included depression, anxiety, disorders, schizophrenia, and dysautonomia that women experienced before pregnancy.
Postnatal depression	1 month after delivery	Postnatal depression was assessed by the Edinburgh Postnatal Depression Scale (EPDS)*.

* The EPDS consists of 10 items rated on a 4-point scale (0−3)[1]. We defined an EPDS score ≥9 as indicating postnatal depression; this has been suggested as the optimal cut-off for the Japanese populations, and its validity and reliability have been reported elsewhere[2-4].

[1] Cox JL, Holden JM, Sagovsky R (1987) Detection of postnatal depression. Development of the 10-item Edinburgh Postnatal Depression Scale. Br J Psychiatry 150:782-786. doi:10.1192/bjp.150.6.782

[2] Harris B, Huckle P, Thomas R, Johns S, Fung H (1989) The use of rating scales to identify post-natal depression. Br J Psychiatry 154:813-817. doi:10.1192/bjp.154.6.813

[3] Murray L, Carothers AD (1990) The validation of the Edinburgh Post-natal Depression Scale on a community sample. Br J Psychiatry 157:288-290. doi:10.1192/bjp.157.2.288

[4] Okano T, Murata M, Masuji F, Tamaki R, Nomura J, Miyaoka H, Kitamura T (1996) Validation and reliability of Japanese version of EPDS (Edinburgh Postnatal Depression Scale). Archives of Psychiatric Diagnostics and Clinical Evaluation 7:525-533
