# Supplementary material for: Association of fish intake with menstrual pain: A cross-sectional study of the Japan Environment and Children’s Study
Source: PLoS One. 2022 Jul 21;17(7):e0269042. doi: 10.1371/journal.pone.0269042 (PMC9302766; doi:10.1371/journal.pone.0269042)
Supplement: S2 Table — (PDF) [file pone.0269042.s002.pdf]

S2 Table. Baseline characteristics according to fish preference

	Preference of fish			
	Likes	Neutral	Dislike	*p* value *
	1593(77.3)	333(16.2)	134(6.5)	
Age (years)				
Mean(S.D)	31.8(5.1)	32.4(5.0)	31.9(5.0)	0.13
≤24	116(7.3)	19(5.7)	8(6.0)	0.52
25 –29	403(25.3)	76(22.8)	33(24.6)	
30 –34	532(33.4)	117(35.1)	49(36.6)	
35–39	399(25.1)	88(26.4)	28(20.9)	
≥40	103(6.5)	27(8.1)	9(6.7)	
Missing	40(2.5)	6(1.8)	7(5.2)	
BMI (kg/m^2)				
<18.5	171(10.7)	43(12.9)	17(12.7)	0.57
18.5–24.9	1089(68.4)	219(65.8)	93(69.4)	
≥25	249(15.6)	58(17.4)	16(11.9)	
Missing	84(5.3)	13(3.9)	8(6.0)	
Smoking habit				
non-smoker	1387(87.1)	281(84.4)	108(80.6)	0.15
Current smoker	169(10.6)	45(13.5)	23(17.2)	
Missing	37(2.3)	7(2.1)	3(2.2)	
Passive smoking				
non-smoker	855(53.7)	174(52.3)	76(56.7)	0.37
Current smoker	688(43.2)	142(42.6)	52(38.8)	
Missing	50(3.1)	17(5.1)	6(4.5)	
Alcohol intake				
None	1053(66.1)	228(68.4)	101(75.4)	0.24
Current drinker	513(32.2)	101(30.3)	31(23.1)	
Missing	27(1.7)	4(1.2)	2(15)	
Maternal educational level				
Junior high school	76(4.8)	15(4.5)	3(2.2)	0.53
High school	675(42.4)	140(42.0)	64(47.8)	
College	822(51.6)	172(51.7)	67(50.0)	
Missing	20(1.3)	6(1.8)	0(0.0)	
Paternal educational level				

Junior high school	102(6.4)	22(6.6)	14(10.5)	0.54
High school	812(51.0)	177(53.2)	64(47.8)	
College	658(41.3)	128(38.4)	55(41.0)	
Missing	21(1.3)	6(1.8)	1(0.8)	
Employment				
Homemaker	763(47.9)	152(45.7)	49(36.6)	0.02
Worker	753(47.3)	167(50.2)	71(53.7)	
Missing	77(4.8)	14(4.2)	13(9.7)	
Family income (×10^4 JPY)				
≤199	73(4.6)	19(5.7)	5(3.7)	0.61
200–399	578(36.3)	112(33.6)	59(44.0)	
400–599	457(28.7)	93(27.9)	30(22.4)	
≥600	348(21.9)	76(22.8)	28(20.9)	
Missing	137(8.6)	33(9.9)	12(9.0)	
Marital status				
Married	1515(95.1)	318(95.5)	125(93.3)	0.48
Others	71(4.5)	15(4.5)	9(6.7)	
Missing	7(0.4)	0(0.0)	0(0.0)	
Parity				
Primipara	573(36.0)	121(36.3)	49(36.6)	0.33
Multipara	1000(62.8)	203(61.0)	84(62.7)	
Missing	20(1.3)	9(2.8)	1(0.8)	
Fetal number				
Singleton	1584(99.4)	331(99.4)	132(98.5)	0.43
Multiple	9(0.6)	2(0.6)	2(1.5)	
Mode of delivery				
Transvaginal	1307(82.1)	274(82.3)	111(82.8)	0.97
Caesarean	286(18.0)	59(17.7	23(17.2)	
Obstetric complications				
None	919(57.7)	176(52.9)	70(52.2)	0.35
Yes	668(41.9)	156(46.9)	64(47.8)	
Missing	6(0.4)	1(0.3)	0(0.0)	
Age at menarche				
≤11	516(32.4)	96(28.8)	46(34.3)	0.20
12-13	755(47.4)	178(53.5)	57(42.5)	
≥14	284(17.8)	51(15.3)	30(22.4)	

Missing	38(2.4)	8(2.4)	1(0.8)	
History of gynecological disease				
No	1477(92.7)	309(92.8)	121(90.3)	0.78
Yes	114(7.2)	24(7.2)	13(9.7)	
missing	2(0.1)	0(0.0)	0(0.0)	
History of mental illness				
No	1471(92.3)	299(89.8)	123(91.8)	0.52
Yes	120(7.5)	34(10.2)	11(8.2)	
Missing	2(0.1)	0(0.0)	0(0.0)	
Postnatal depression (EPDS ≥9 points)				
No	1361(85.4)	273(82.0)	100(74.6)	0.01
Yes	200(12.6)	53(15.9)	29(21.6)	
Missing	32(2.0)	7(2.1)	5(3.7)	
Menstrual pain				
No pain	300(18.8)	57(17.1)	18(13.4)	0.002
Mild	852(53.5)	184(55.3)	70(52.2)	
Moderate	390(24.5)	81(24.3)	32(23.9)	
Severe	51(3.2)	11(3.3)	14(10.5)	

* Calculated using chi-square tests for categorical variables or a one-way ANOVA for continuous normally distributed variables.

SD, standard deviation; BMI, body mass index; JPY, Japanese yen; EPDS, Edinburgh Postnatal Depression Scale; ANOVA, analysis of variance
