# Supplementary material for: Association of fish intake with menstrual pain: A cross-sectional study of the Japan Environment and Children’s Study
Source: PLoS One. 2022 Jul 21;17(7):e0269042. doi: 10.1371/journal.pone.0269042 (PMC9302766; doi:10.1371/journal.pone.0269042)
Supplement: S3 Table — (PDF) [file pone.0269042.s003.pdf]

S3 Table. Baseline characteristics according to smoking habit or parity

	Smoking habit			Parity		
	No	Yes	*p* value *	Primipara	Multipara	*p* value *
	1776(86.2)	237(11.5)		743(36.1)	1287(62.5)	
Age (years)						
Mean(S.D)	32.1(4.9)	30.9(5.5)	0.003	30.2(5.2)	32.9(4.7)	<0.001
≤24	106(6.0)	32(13.5)	<0.001	94(12.7)	45(3.5)	<0.001
25 –29	435(24.5)	65(27.4)		248(33.4)	257(20.0)	
30 –34	622(35.0)	69(29.1)		216(29.1)	469(36.4)	
35–39	467(26.3)	43(18.1)		124(16.7)	387(30.1)	
≥40	121(6.8)	15(6.3)		40(5.4)	97(7.5)	
Missing	25(1.4)	13(5.5)		21(2.8)	32(2.5)	
BMI (kg/m^2)						
<18.5	197(11.1)	30(12.7)	<0.001	92(12.4)	134(10.4)	0.56
18.5–24.9	1236(69.6)	135(57.0)		500(67.3)	881(68.5)	
25≤	261(14.7)	55(23.2)		108(14.5)	211(16.4)	
Missing	82(4.6)	17(7.2)		43(5.8)	61(4.7)	
Smoking habit						
non-smoker				655(88.2)	1093(84.9)	0.07
Current smoker				69(9.3)	167(13.0)	
Missing				19(2.6)	27(2.1)	

Passive smoking						
non-smoker	1079(60.8)	23(9.7)	<0.001	419(56.4)	671(52.1)	0.18
Current smoker	669(37.7)	211(89.0)		294(39.6)	575(44.7)	
Missing	28(1.6)	3(1.3)		30(4.0)	41(3.2)	
Alcohol intake						
None	1243(70.0)	130(54.9)	<0.001	526(70.8)	830(64.5)	0.001
Current drinker	532(30.0)	107(45.2)		202(27.2)	440(34.2)	
Missing	1(0.1)	0(0.0)		15(2.0)	17(1.3)	
Maternal educational level						
Junior high school	50(2.8)	35(14.8)	<0.001	27(3.6)	67(5.2)	0.34
High school	728(41.0)	135(57.0)		310(41.7)	558(43.4)	
College	976(55.0)	64(27.0)		397(53.4)	645(50.1)	
Missing	22(1.2)	3(1.3)		9(1.2)	17(1.3)	
Paternal educational level						
Junior high school	88(5.0)	43(18.1)	<0.001	39(5.3)	98(7.6)	0.44
High school	887(49.9)	141(59.5)		388(52.2)	650(50.5)	
College	779(43.9)	49(20.7)		305(41.1)	523(40.6)	
Missing	22(1.2)	4(1.7)		11(1.5)	16(1.2)	
Employment						
Homemaker	847(47.7)	103(43.5)	0.05	355(47.8)	593(46.1)	0.67
Worker	859(48.4)	117(49.4)		346(46.6)	633(49.2)	
missing	70(3.9)	17(7.2)		42(5.7)	61(4.7)	

Family income (×10⁴ JPY)						
≤199	74(4.2)	18(7.6)	0.006	32(4.3)	63(4.9)	0.3
200–399	644(36.3)	96(40.5)		275(37.0)	465(36.1)	
400–599	502(28.3)	66(27.9)		203(27.3)	370(28.8)	
600≤	404(22.8)	33(13.9)		157(21.1)	289(22.5)	
Missing	152(8.6)	24(10.1)		76(10.2)	100(7.8)	
Marital status						
Married	1701(95.8)	214(90.3)	<0.001	668(89.9)	1264(98.2)	<0.001
Others	69(3.9)	22(9.3)		71(9.6)	21(1.6)	
Missing	6(0.3)	1(0.4)		4(0.5)	2(0.2)	
Parity						
Primipara	655(36.9)	69(29.1)	0.02			
Multipara	1093(61.5)	167(70.5)				
Missing	28(1.6)	1(0.4)				
Fetal number						
Singleton	1765(99.4)	235(99.2)	0.69	736(99.1)	1282(99.6)	0.05
Multiple	11(0.6)	2(0.8)		7(0.9)	5(0.4)	
Mode of delivery						
Transvaginal	1455(81.9)	198(83.5)	0.54	605(81.4)	1063(82.6)	0.77
Caesarean	321(18.1)	39(16.5)		138(18.6)	224(17.4)	
Obstetric complications						
None	1022(57.6)	116(49.0)	0.02	395(53.2)	759(59.0)	0.02

Yes	747(42.1)	121(51.1)		346(46.6)	523(40.6)	
Missing	7(0.4)	0(0.0)		2(0.3)	5(0.4)	
Age at menarche						
≤11	567(31.9)	80(33.8)	0.40	221(29.7)	426(33.1)	0.08
12-13	851(47.9)	117(49.4)		358(48.2)	618(48.0)	
≥14	320(18.0)	33(13.9)		140(18.8)	220(17.1)	
Missing	38(2.1)	7(3.0)		24(3.2)	23(1.8)	
History of gynecological disease						
No	1639(92.3)	225(94.9)	0.32	682(91.8)	1200(93.2)	0.11
Yes	135(7.6)	12(5.1)		59(7.9)	87(6.8)	
Missing	2(0.1)	0(0.0)		2(0.3)	0(0.0)	
History of mental illness						
No	1640(92.3)	212(89.5)	0.24	681(91.7)	1185(92.1)	0.44
Yes	134(7.6)	25(10.6)		60(8.1)	102(7.9)	
Missing	2(0.1)	0(0.0)		2(0.3)	0(0.0)	
Postnatal depression (EPDS ≥9 points)						
No	1514(85.3)	182(76.8)	0.003	582(78.3)	1128(87.7)	<0.001
Yes	229(12.9)	47(19.8)		146(19.7)	131(10.2)	
Missing	33(1.9)	8(3.4)		15(2.0)	28(2.2)	

* Calculated using chi-square tests for categorical variables or a one-way ANOVA for continuous normally distributed variables.

SD, standard deviation; BMI, body mass index; JPY, Japanese yen; EPDS, Edinburgh Postnatal Depression Scale; ANOVA, analysis of variance
