# Supplementary material for: Association of fish intake with menstrual pain: A cross-sectional study of the Japan Environment and Children’s Study
Source: PLoS One. 2022 Jul 21;17(7):e0269042. doi: 10.1371/journal.pone.0269042 (PMC9302766; doi:10.1371/journal.pone.0269042)
Supplement: Attachment — Submitted filename: Response_to_Reviewers.docx [file pone.0269042.s004.docx]

Dear Editors and Reviewers

Thank you very much for reviewing our manuscript and for offering valuable advice. We have addressed your comments with point-by-point responses and revised the manuscript accordingly.

Responses to the Comments by the reviewer 1:

Thank you very much for taking the time to point out a number of critical points.

Title
Authors can indicate that it is a cross-sectional study

**Response**: Thank you for your kind suggestion. We have modified the "Title" to

*“Association of fish intake with menstrual pain: A cross-sectional study of the Japan Environment and Children’s Study”*

- Please prepare a list of abbreviations at the beginning of the Meta DATA.

**Response**: Thank you for your helpful recommendation. We have added a list of abbreviations before the "Abstract" section.

Abstracts
1) Abstract should be informative, background did not explain the question of this review and the answer which authors search for it

**Response**: Thank you very much for your invaluable comments. As you importantly pointed out, the background of the Abstract was not adequately described. Therefore, we have enriched the background as follows.

*“The relationship between fish eating habits and menstrual pain is unknown. Elucidating this relationship can inform dietary guidance for reproductive age women with menstrual pain.” (Page 4, Lines 36–38).*

2) Keywords: are these keywords are Mesh terms? Word that serves as a keyword, as to the meaning of that condition must be a Mesh term

**Response**: Thank you for your helpful recommendation. We have modified the Keywords to Mesh terms.

*“dysmenorrhea, menstrual pain, cross-sectional study, feeding behavior.”*

*(On submission system)*

Introduction
The Introduction needs adjustments in order to answer these questions:

- What are the uncertainties and conflicts that underlie the hypotheticals?

- What is the focused clinical question your research will address?

**Respons**e: Thank you very much for your invaluable comments. We apologize for the insufficient information about uncertainties in previous reports and clinical questions. The clinical question is whether fish consumption is effective for dysmenorrhea. As you have mentioned, we have supplemented the "Introduction" section.

*“Thus, it is uncertain whether fish consumption is effective for dysmenorrhea as previous studies have not yielded consistent results due to small sample sizes.” (Pages 6–7, Lines 86–88)*

- How important is the evidence of studies for the healthy individuals and patients?

**Response**: Thank you for pointing this out. As you mentioned, there was a lack of description of the importance of our study to healthy individuals and patients. Thus, I have added the following description in the Introduction section.

*“Elucidating this relationship is informative because it can inform dietary guidance for reproductive age women with menstrual pain and may have a positive impact on many aspects of personal life. In addition, this relationship may be useful to healthy individuals in their daily food choices.” (Page 7, Lines 89–93)*

Discussion
- The authors should list and shortly discuss the limitation of their study, for instance their limited number of participants and small sample size and also variation between type of fish that may affect the results

**Response**: Thank you very much for your important comments. As you pointed out, there are limitations regarding the limited number of patients and small sample size. Therefore, we have modified the Discussion section to include this limitation.

*“Second, the sample size was relatively small; thus, our findings should be interpreted with caution.” (Page 24, Lines 324–325)*

Also, as you mentioned, our questionnaire did not distinguish variation between type of fish, which may have affected the results. We have modified the text in the Discussion section, as follows:

“Third, this study assessed only fish intake frequency and may not accurately reflect fish consumption or type of fish; therefore, we could not assess the total intake of each nutrient, including n-3 fatty acids.” *(Page 24, Lines 325–327)*

In addition, the strength of our study lies in the ability to adjust for many covariates. Thus, we modified as the introductory paragraph of the Discussion follows:

*“In the present study, we adjusted for many covariates, and only few studies have been able to adjust for such a large number covariates to examine the associations between fish intake frequency and the risk of menstrual pain. ~~To our knowledge, this is the first study performed with adequate meaningful power to examine associations between fish intake frequency and the risk of menstrual pain.”~~ (Page 20, Lines 261–265)*

Responses to the Comments by the reviewer 2:

1. The fish intake before delivery or when she was little is unknown, and I think that this point is more important to investigate the association between fish consumption and dysmenorrhea. It must be described as a big limitation. However, it is epidemiologically interested the results and your discussion about the mechanism.

**Response**: Thank you very much for your important comments. Our study does not have data on fish intake frequency before delivery. As you importantly pointed out, whether long-term fish intake is associated with dysmenorrhea or a short-term effect could not be demonstrated in this study and is a limitation of the present study. Therefore, we have modified the Discussion section to include this limitation.

*“In addition, the influence of non-reported food items, caloric intake, or prenatal fish intake could not be excluded.” (Page 24, Lines 330–331).*

Again, thank you for giving us the opportunity to strengthen our manuscript with your valuable comments and queries. We have worked hard to incorporate your feedback and hope that these revisions persuade you to accept our submission.
